# Risk factors for the failure of first‐line PARP inhibitor maintenance therapy in patients with advanced ovarian cancer: Gynecologic Oncology Research Investigators Collaboration Study (GORILLA‐3004)

**DOI:** 10.1002/cam4.6546

**Published:** 2023-09-28

**Authors:** Nam Kyeong Kim, Yeorae Kim, Hee Seung Kim, Soo Jin Park, Dong Won Hwang, Sung Jong Lee, Ji Geun Yoo, Suk‐Joon Chang, Joo‐Hyuk Son, Tae‐Wook Kong, Jeeyeon Kim, Seung‐Hyuk Shim, A Jin Lee, Dong Hoon Suh, Yoo‐Young Lee

**Affiliations:** ^1^ Department of Obstetrics and Gynecology Seoul National University Bundang Hospital Seongnam Korea; ^2^ Department of Obstetrics and Gynecology Seoul National University College of Medicine Seoul Korea; ^3^ Department of Obstetrics and Gynecology Seoul National University Hospital Seoul Korea; ^4^ Department of Obstetrics and Gynecology Seoul St. Mary's Hospital Seoul Korea; ^5^ Department of Obstetrics and Gynecology The Catholic University of Korea Seoul Korea; ^6^ Department of Obstetrics and Gynecology, Daejeon St. Mary's Hospital The Catholic University of Korea Daejeon Korea; ^7^ Department of Obstetrics and Gynecology Ajou University School of Medicine Suwon Korea; ^8^ Department of Obstetrics and Gynecology, Research Institute of Medical Science Konkuk University School of Medicine Seoul Korea; ^9^ Department of Obstetrics and Gynecology, Samsung Medical Center Sungkyunkwan University School of Medicine Seoul Korea

**Keywords:** first‐line maintenance therapy, ovarian cancer, poly (ADP‐ribose) polymerase inhibitor, recurrence, risk factor

## Abstract

**Objective:**

To identify the risk factors for failure of first‐line poly (ADP‐ribose) polymerase inhibitor (PARPi) maintenance therapy in patients with advanced ovarian cancer.

**Method:**

Patients with stage III‐IV epithelial ovarian cancer who received first‐line PARPi maintenance therapy were retrospectively reviewed. Clinicopathologic factors were compared between two groups—recur/progression of disease (PD) and non‐recur/PD.

**Results:**

In total, 191 patients were included. Median follow‐up was 9.9 months, and recurrence rate was 20.9%. *BRCA* mutations were found in 63.4% patients. Postoperative residual tumor (60.5% vs. 37.8%), non‐high grade serous carcinoma (HGSC) (15.0% vs. 6.0%), neoadjuvant chemotherapy (NAC) (55.0% vs. 35.8%), and pre‐PARPi serum CA‐125 levels ≥23.5 U/mL (35.9% vs. 15.2%) were more frequently observed in the recur/PD group. Multivariate Cox‐regression analysis revealed pre‐PARPi serum CA‐125 levels ≥23.5 U/mL (HR, 2.17; 95%CI, 1.03–4.57; *p* = 0.042), non‐HGSC (3.28; 1.20–8.97; *p* = 0.021), NAC (2.11; 1.04–4.26; *p* = 0.037), and no *BRCA* mutation (2.23; 1.12–4.44; *p* = 0.023) as independent risk factors associated with poor progression‐free survival (PFS). A subgroup analysis according to *BRCA* mutation status showed that pre‐PARPi serum CA‐125 levels ≥26.4 U/mL were the only independent risk factor for poor PFS in women with *BRCA* mutations (2.75; 1.03–7.39; *p* = 0.044). Non‐HGSC (5.05; 1.80–14.18; *p* = 0.002) and NAC (3.36; 1.25–9.04; *p* = 0.016) were independent risk factors in women without *BRCA* mutations.

**Conclusion:**

High pre‐PARPi serum CA‐125 levels, non‐HGSC histology, NAC, and no *BRCA* mutation might be risk factors for early failure of first‐line PARPi maintenance therapy. In women with *BRCA* mutations, high pre‐PARPi serum CA‐125 levels, which represent a large tumor burden before PARPi, were the only independent risk factor for poor PFS.

## 1 INTRODUCTION

Ovarian cancer is known to be the most lethal gynecological cancer, with a 5‐year survival rate of <50% for advanced disease.^1^ Approximately 70% of ovarian cancer patients are initially diagnosed at advanced stage.^2^ Despite adjuvant platinum‐based chemotherapy after cytoreductive surgery being the standard treatment,^3^, ^4^ 75% of stage IIB‐IV ovarian cancer patients eventually experience a relapse, which results in poor survival outcomes.^5^ There has long been an unmet need to prevent recurrence and maximize progression‐free survival (PFS) after completion of front‐line treatments in advanced ovarian cancer.

As the concept of maintenance treatment for ovarian cancer has emerged to reduce the risk of recurrence, randomized controlled trials of poly (ADP‐ribose) polymerase inhibitors (PARPi) as first‐line maintenance treatment in advanced ovarian cancer have shown promising results. A study on olaparib as a first‐line maintenance treatment after platinum‐based chemotherapy (SOLO‐1 trial) showed significant PFS and overall survival (OS) benefits in patients with newly diagnosed advanced ovarian cancer and *BRCA* mutations.^6^, ^7^ A study using another PARPi, niraparib, as a first‐line maintenance therapy (PRIMA trial) reported significantly increased PFS regardless of homologous recombination status.^8^ Based on these results, the National Comprehensive Cancer Network and the European Society for Medical Oncology recommend PARPi maintenance treatment for advanced ovarian cancer patients who have a complete or partial response to first‐line platinum‐based chemotherapy.^3^, ^9^

Although first‐line PARPi maintenance has been used worldwide for a long time, failure after first‐line PARPi maintenance treatment has been reported, and the issue of PARPi resistance has recently emerged.^10^, ^11^ To further enhance treatment efficacy, recent studies have focused on resistance to PARPi, combination treatment with other targeted agents or immunotherapy to overcome PARPi resistance, and PARPi retreatment in patients with recurrence after using PARPi.^12^, ^13^ However, it is important to understand the risk factors for recurrence after first‐line PARPi maintenance treatment to select an appropriate patient group for PARPi use and to establish an optimal treatment plan for patients with newly diagnosed advanced ovarian cancer. As the duration of PARPi use as first‐line maintenance treatment in a real‐world clinical setting is relatively short, there are no studies evaluating the outcomes and risk factors for recurrence in patients using first‐line PARPi maintenance treatment for advanced ovarian cancer.

This multicenter retrospective study aimed to identify the risk factors for the failure of first‐line PARPi maintenance therapy in patients with advanced ovarian cancer.

## 2 METHODS

This retrospective study was conducted at six university hospitals in Korea, and all institutions were approved by the Institutional Review Board. The requirement for obtaining informed consent from the patients was waived because the study was based on retrospective review of medical charts. The medical records of consecutive patients who received first‐line PARPi maintenance therapy for advanced ovarian cancer from January 2018 to June 2022 were retrospectively reviewed. The inclusion criteria were as follows: (1) patients diagnosed with International Federation of Gynecology and Obstetrics (FIGO) stage III‐IV and histologically confirmed epithelial ovarian, tubal, or primary peritoneal cancer; (2) patients who used PARPi as first‐line maintenance treatment. Patients who were diagnosed and treated for cancers other than breast cancer and endometrial cancer within the last 5 years were excluded.

Information about clinical characteristics, including age at diagnosis, the body mass index, parity, initial serum CA‐125 levels at diagnosis, histologic type, FIGO stage, primary treatment for ovarian cancer, residual tumor after staging surgery, neoadjuvant and adjuvant chemotherapy, and serum CA‐125 levels before starting PARPi, was collected. Data on PARPi treatment, including type, duration, discontinuation, and reason for discontinuation, were also collected. Cutoff values for initial CA‐125 at diagnosis and CA‐125 before starting PARPi were set as the mean values for the overall study population, patients with BRCA mutations, and patients without BRCA mutations, respectively. *BRCA* mutations in tumor tissue or blood were retrospectively reviewed, and subgroup analyses were performed according to *BRCA* mutation status. PFS was defined as the time from the initial diagnosis of cancer to disease progression, based on the Response Evaluation Criteria in Solid Tumors (RECIST) for imaging evaluation, or death from any cause.

Clinicopathological factors were compared between patients who experienced recurrence or progression of disease (PD) during PARPi maintenance (recur/PD group) and those who did not (non‐recur/PD group) using Student's *t*‐test and the *χ* ^2^ test. Univariate and multivariate Cox regression analyses were conducted to identify the risk factors for short PFS after first‐line PARPi maintenance therapy. Statistical analyses were performed using IBM SPSS Statistics for Windows (version 25.0; IBM Corp.). Statistical significance was set at *p* < 0.05.

## 3 RESULTS

A total of 191 patients who met the inclusion criteria were enrolled in this study. Median follow‐up period from the start of PARPi was 9.9 months (range, 0.9–30.9 months), and the recurrence rate was 20.9% (40/191). The baseline characteristics of the study population are shown in Table 1. The frequency of CA‐125 abnormalities (i.e., > 35 U/mL) before starting PARPi was 13.2% (25/190). *BRCA* mutations were found in 121 patients (63.4%), with 81 showing *BRCA1*, and 41 showing *BRCA2* mutations. One patient had mutations in both *BRCA1* and *BRCA 2*. Seventy‐seven (40.3%) and 114 (59.7%) patients took olaparib and niraparib, respectively. The median duration of PARPi use was 8.5 months (range, 0.9–30.4 months). Of 40 patients in the recur/PD group, only 2 (5.0%) stopped PARPi before recur/PD because of adverse events and 38 (95.0%) continued PARPi until PD. One patient in the non‐recur/PD group discontinued PARPi owing to myelodysplastic syndrome.

**TABLE 1:** Baseline characteristics of the study population (*N* = 191).

Variable	Value
Age at diagnosis (years)	57.1 ± 10.0
BMI	22.9 ± 3.3
Parity	2.0 ± 1.0
Pretreatment CA‐125 (U/mL)	1649.0 ± 2930.5
FIGO stage	
IIIA	6 (3.1)
IIIB	12 (6.3)
IIIC	90 (47.1)
IVA	11 (5.8)
IVB	72 (37.7)
Histology	
HGSC	176 (92.1)
Endometrioid carcinoma	3 (1.6)
Clear cell carcinoma	7 (3.7)
Carcinosarcoma	3 (1.6)
Mixed	2 (1.0)
Primary treatment	
PDS	115 (60.2)
NAC followed by IDS	71 (37.2)
Palliative chemotherapy only	5 (2.6)
Residual disease after debulking surgery	
No residual	107 (56.0)
< 1 cm	58 (30.4)
≥ 1 cm	21 (11.0)
Not available	5 (2.6)
The number of total chemotherapy cycles before PARPi	
<6	3 (1.6)
6–8	155 (81.2)
9–11	29 (15.2)
≥12	4 (2.1)
Other maintenance treatment before PARPi use	5 (2.6)
CA‐125 before starting PARPi (U/mL)	23.5 ± 59.3
Type of PARPi	
Olaparib	77 (40.3)
Niraparib	114 (59.7)
Discontinuation of PARPi	49 (25.7)
Reason of PARPi discontinuation^a^	
End of planned 2‐year treatment	4 (8.2)
Recurrence or PD	38 (77.6)
Adverse events	6 (12.2)
Ileus	1 (2.0)
*BRCA* mutation	
*BRCA1*	81 (42.4)
*BRCA2*	41 (21.5)

*Note*: Values are presented as mean ± standard deviation or number (%).

Abbreviations: BMI, body mass index; FIGO, International Federation Of Gynecology And Obstetrics; HGSC, high‐grade serous carcinoma; IDS, interval debulking surgery; NAC, neoadjuvant chemotherapy; PARPi, poly (ADP‐ribose) polymerase inhibitor; PD, progression of disease; PDS, primary debulking surgery.

^a^ A total of 49 patients stopped PARPi at the time of data collection.

Clinicopathological factors were compared between the recur/PD and non‐recur/PD groups (Table 2). Postoperative gross residual tumor (60.5% [23/38] vs. 37.8% [56/148]; *p* = 0.012), neoadjuvant chemotherapy (NAC) followed by interval debulking surgery (IDS) as primary treatment (55.0% [22/40] vs. 35.8% [54/151]; *p* = 0.027), and high serum CA‐125 levels before starting PARPi ≥23.5 U/mL (35.9% [14/39] vs. 15.2% [23/151]; *p* = 0.004) were more frequently observed in the recur/PD group than in the non‐recur/PD group. The frequency of CA‐125 abnormalities before starting PARPi was also significantly higher in the recur/PD group compared to the non‐recur/PD group (28.2% [11/39] vs. 9.3% [14/151]; *p* = 0.002). *BRCA* mutation rate was lower in the recur/PD group than in the non‐recur/PD group (50.0% [20/40] vs. 66.9% [101/151]; *p* = 0.049). There were no differences in age at diagnosis, initial CA‐125 levels at diagnosis, total number of chemotherapy cycles before PARPi use, FIGO stage, tumor histology (high‐grade serous carcinoma [HGSC] vs. non‐HGSC), and type of PARPi between the two groups.

**TABLE 2:** Clinicopathologic factors according to cancer recurrence or progression.

Variable	Non‐recur/PD group (*n* = 151)	Recur/PD group (*n* = 40)	*p*
Age at diagnosis (years)	56.9 ± 9.9	58.2 ± 10.4	0.441
< 57	77 (51.0)	18 (45.0)	0.500
≥ 57	74 (49.0)	22 (55.0)	
Initial CA‐125 at diagnosis (U/mL)	1726.9 ± 3165.2	1347.3 ± 1745.7	0.472
< 1649.0	114 (75.5)	30 (76.9)	0.853
≥ 1649.0	37 (24.5)	9 (23.1)	
CA‐125 before PARPi (U/mL)	20.7 ± 61.0	34.2 ± 51.0	0.205
< 23.5	128 (84.8)	25 (64.1)	0.004
≥ 23.5	23 (15.2)	14 (35.9)	
Total number of chemotherapy cycles before PARPi	6.7 ± 1.5	6.8 ± 1.7	0.665
FIGO stage			0.194
III	89 (58.9)	19 (47.5)	
IV	62 (41.1)	21 (52.5)	
Postoperative gross residual disease			0.012
No	92 (62.2)	15 (39.5)	
Yes	56 (37.8)	23 (60.5)	
Histology			0.059
HGSC	142 (94.0)	34 (85.0)	
Non‐HGSC	9 (6.0)	6 (15.0)	
Type of PARPi			0.257
Olaparib	64 (42.4)	13 (32.5)	
Niraparib	87 (57.6)	27 (67.5)	
Duration of PARPi use (months)	11.0 ± 7.2	6.3 ± 4.7	<0.001
Non‐recur discontinuation of PARPi^a^	9 (100.0)	2 (5.0)	<0.001
Primary treatment			0.027
PDS	97 (64.2)	18 (45.0)	
NAC followed by IDS^b^	54 (35.8)	22 (55.0)	
*BRCA* mutation	101 (66.9)	20 (50.0)	0.049
*BRCA1*	66 (43.7)	15 (37.5)	0.480
*BRCA2*	35 (23.2)	6 (15.0)	0.263

*Note*: Values are presented as mean ± standard deviation or number (%).

Abbreviations: FIGO, International Federation Of Gynecology And Obstetrics; HGSC, high‐grade serous carcinoma; IDS, interval debulking surgery; NAC, neoadjuvant chemotherapy; PARPi, Poly (ADP‐ribose) polymerase inhibitor; PD, progression of disease; PDS, primary debulking surgery.

^a^ Including 4 (end of planned 2‐year treatment) and 7 (adverse event).

^b^ Five patients who underwent palliative chemotherapy without surgery were included.

The results of univariate and multivariate Cox regression analyses of risk factors for short PFS are shown in Table 3. The multivariate Cox‐regression analysis revealed that serum CA‐125 levels before starting PARPi ≥23.5 U/mL (HR, 2.17; 95% CI, 1.03–4.57; *p* = 0.042), non‐HGSC (HR, 3.28; 95% CI, 1.20–8.97; *p* = 0.021), NAC followed by IDS (HR, 2.11; 95% CI, 1.04–4.26; *p* = 0.037), and no *BRCA* mutation (HR, 2.23; 95% CI, 1.12–4.44; *p* = 0.023) were independent risk factors associated with a poor PFS.

**TABLE 3:** Univariate and multivariate Cox regression analyses of risk factors for progression‐free survival.

Variable	*N* (%)	Univariate			Multivariate		
		HR	95% CI	*p*	HR	95% CI	*p*
Age at diagnosis (years)							
< 57	95 (49.7)	1					
≥ 57	96 (50.3)	1.23	0.65–2.33	0.523			
Pretreatment CA‐125 (U/ml)							
< 1649.0	144 (75.8)	1					
≥ 1649.0	46 (24.2)	0.99	0.47–2.11	0.984			
CA‐125 before starting PARPi (U/ml)							
< 23.5	153 (80.5)	1			1		
≥ 23.5	37 (19.5)	2.05	1.03–4.09	0.042	2.17	1.03–4.57	0.042
FIGO stage							
III	108 (56.5)	1					
IV	83 (43.5)	1.35	0.71–2.56	0.364			
Tumor histologic type							
HGSC	176 (92.1)	1			1		
Non‐HGSC	15 (7.9)	3.07	1.27–7.42	0.013	3.28	1.20–8.97	0.021
Residual disease							
No	107 (57.5)	1					
Yes	79 (42.5)	1.80	0.92–3.49	0.084			
The number of total chemotherapy cycles before PARPi							
≤ 6	144 (75.4)	1					
>6	47 (24.6)	1.10	0.55–2.22	0.789			
Type of PARPi							
Olaparib	77 (40.3)	1					
Niraparib	114 (59.7)	1.81	0.92–3.59	0.087			
Primary treatment							
PDS	115 (60.2)	1			1		
No PDS^a^	76 (39.8)	2.04	1.08–3.87	0.029	2.11	1.04–4.26	0.037
*BRCA* mutation							
Yes	121 (63.4)	1			1		
No	70 (36.6)	2.41	1.27–4.60	0.007	2.23	1.12–4.44	0.023

Abbreviations: FIGO, International Federation of Gynecology and Obstetrics; HGSC, high‐grade serous carcinoma; IDS, interval debulking surgery; NAC, neoadjuvant chemotherapy; PARPi, Poly (ADP‐ribose) polymerase inhibitor; PD, progression of disease; PDS, primary debulking surgery.

^a^ Including neoadjuvant chemotherapy and palliative chemotherapy.

**FIGURE 1:**
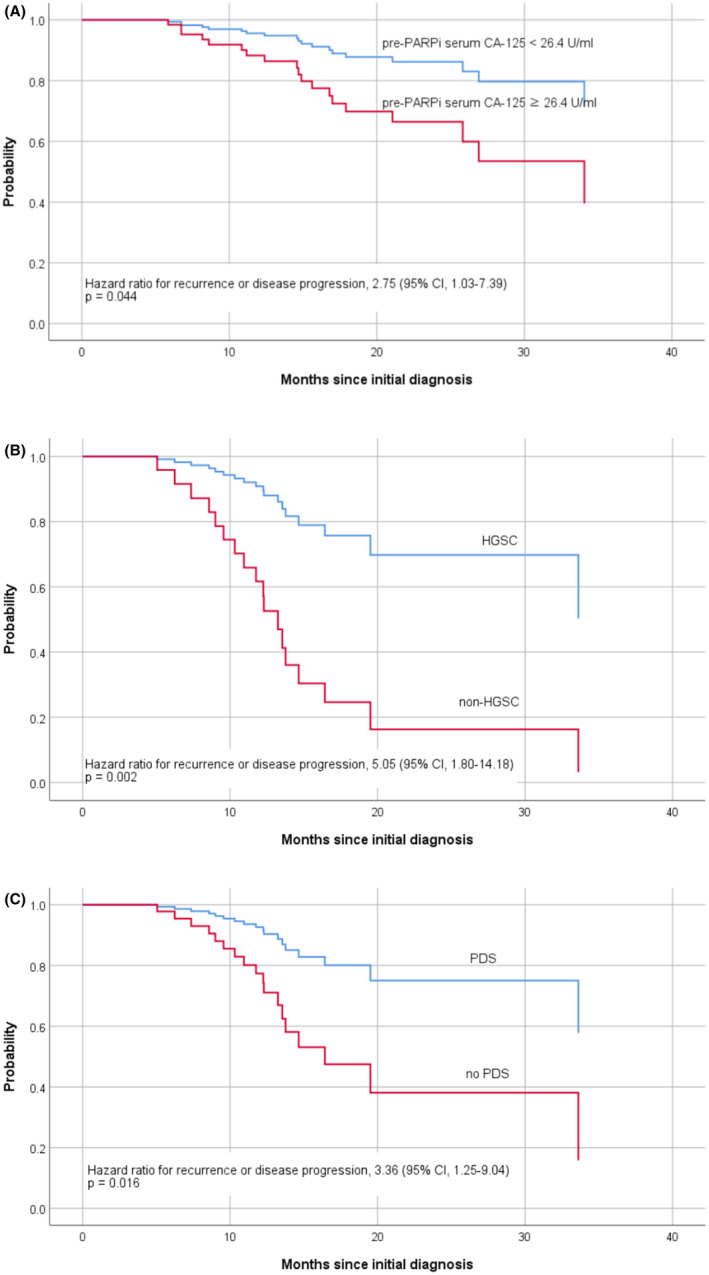
Progression‐free survival in patients with *BRCA* mutation according to pre‐PARPi serum CA125 levels (A) and in patients without *BRCA* mutation according to histologic type, (B) and primary treatment, (C). PARPi, poly (ADP‐ribose) polymerase inhibitor; HGSC, high‐grade serous carcinoma; PDS, primary debulking surgery; NAC, neoadjuvant chemotherapy.

We performed a subgroup analysis according to *BRCA* mutation status. In patients with *BRCA* mutations, the recurrence rate was 16.5% (20/121); additionally, high serum CA‐125 levels before starting PARPi ≥26.4 U/mL (35.0% [7/20] vs. 14.9% [15/101]; *p* = 0.033) and postoperative gross residual tumor (63.2% [12/19] vs. 36.7% [36/98]; *p* = 0.032) were observed more frequently in the recur/PD group than in the non‐recur/PD group (Table S1). Multivariate Cox‐regression analysis showed that serum CA‐125 levels before starting PARPi ≥26.4 U/mL (HR, 2.75; 95% CI, 1.03–7.39; *p* = 0.044) were the only independent risk factor for poor PFS in women with *BRCA* mutations (Table 4). Kaplan–Meier curves of PFS according to serum CA‐125 levels before starting PARPi are shown in Figure 1A.

**TABLE 4:** Univariate and multivariate Cox regression analyses of risk factors for progression‐free survival in patients with *BRCA* mutation (*N* = 121).

Variable	*N* (%)	Univariate			Multivariate		
		HR	95% CI	*p*	HR	95% CI	*p*
Age at diagnosis (years)							
< 58	62 (51.2)	1					
≥ 58	59 (48.8)	0.86	0.36–2.08	0.734			
Pretreatment CA‐125 (U/mL)							
< 1872.3	89 (73.6)	1			1		
≥ 1872.3	32 (26.4)	1.58	0.63–3.96	0.333	1.31	0.45–3.83	0.621
CA‐125 before PARPi (U/ml)							
< 26.4	99 (81.8)	1			1		
≥ 26.4	22 (18.2)	2.91	1.15–7.39	0.025	2.75	1.03–7.39	0.044
FIGO stage							
III	65 (53.7)	1					
IV	56 (46.3)	1.33	0.55–3.22	0.533			
Tumor histologic type							
HGSC	116 (95.9)	1					
Non‐HGSC	5 (4.1)	0.05	0–1245.16	0.555			
Residual disease							
No	69 (57.0)	1			1		
Yes	48 (39.7)	2.07	0.81–5.31	0.128	1.79	0.69–4.66	0.235
Total cycle number of chemotherapy before PARPi							
≤ 6	89 (73.6)	1					
> 6	32 (26.4)	1.39	0.55–3.50	0.486			
Type of PARPi							
Olaparib	75 (62.0)	1					
Niraparib	46 (38.0)	1.01	0.40–2.57	0.984			
Primary treatment							
PDS	72 (59.5)	1			1		
No PDS^a^	49 (40.5)	1.55	0.64–3.75	0.333	1.25	0.46–3.46	0.662

Abbreviations: FIGO, International Federation of Gynecology and Obstetrics; HGSC, high‐grade serous carcinoma; PARPi, Poly (ADP‐ribose) polymerase inhibitor; PDS, primary debulking surgery.

^a^ Including neoadjuvant chemotherapy and palliative chemotherapy.

The recurrence rate was 28.6% (20/70) in women without *BRCA* mutations. There were more patients with serum CA‐125 levels before starting PARPi ≥18.3 U/mL (31.6% [6/19] vs. 10.0% [5/50]; *p* = 0.029), patients with non‐HGSC (30.0% [6/20] vs. 8.0% [4/50]; *p* = 0.027), and patients who received NAC followed by IDS (60.0% [12/20] vs. 30.0% [15/50]; *p* = 0.020) in the recur/PD group than in the non‐recur/PD group (Table S2). Non‐HGSC (HR, 5.05; 95% CI, 1.80–14.18; *p* = 0.002) and NAC followed by IDS (HR, 3.36; 95% CI, 1.25–9.04; *p* = 0.016) were independent risk factors associated with a short PFS (Table 5). Kaplan–Meier curves of PFS according to histologic type and primary treatment are shown in Figure 1B,C, respectively.

**TABLE 5:** Univariate and multivariate Cox regression analyses of risk factors for progression‐free survival in patients without *BRCA* mutation (*N* = 70).

Variable	*N* (%)	Univariate			Multivariate		
		HR	95% CI	*p*	HR	95% CI	*p*
Age at diagnosis (years)							
< 56	35 (50.0)	1					
≥ 56	35 (50.0)	1.97	0.76–5.12	0.162			
Initial CA‐125 at diagnosis (U/ml)							
< 1257.4	56 (80.0)	1					
≥ 1257.4	13 (18.6)	0.66	0.15–2.91	0.582			
CA‐125 before PARPi (U/ml)							
< 18.3	58 (82.9)	1					
≥ 18.3	11 (15.7)	1.48	0.47–4.68	0.503			
FIGO stage							
III	43 (61.4)	1					
IV	27 (38.6)	1.54	0.59–3.98	0.379			
Tumor histologic type							
HGSC	60 (85.7)	1			1		
Non‐HGSC	10 (14.3)	4.18	1.53–11.40	0.005	5.05	1.80–14.18	0.002
Residual disease							
No	38 (54.3)	1					
Yes	31 (44.3)	1.62	0.60–4.35	0.340			
Total cycle number of chemotherapy before PARPi							
≤ 6	55 (78.6)	1					
> 6	15 (21.4)	0.68	0.20–2.37	0.543			
Type of PARPi							
Olaparib	2 (2.9)	1					
Niraparib	68 (97.1)	21.87	0–422238.00	0.540			
Primary treatment							
PDS	43 (61.4)	1			1		
No PDS^a^	27 (38.6)	2.84	1.08–7.47	0.035	3.36	1.25–9.04	0.016

Abbreviations: FIGO, International Federation of Gynecology and Obstetrics; HGSC, high‐grade serous carcinoma; PARPi, Poly (ADP‐ribose) polymerase inhibitor; PDS, primary debulking surgery.

^a^ Including neoadjuvant chemotherapy and palliative chemotherapy.

## 4 CONCLUSIONS

The present study showed that high serum CA‐125 levels before starting PARPi were the only independent risk factor for short PFS in women with *BRCA* mutation, whereas non‐HGSC (vs. HGSC) and NAC (vs. primary debulking surgery [PDS]) were risk factors in women without *BRCA* mutation. Non‐HGSC histology, NAC, high serum CA‐125 levels before starting PARPi, and no *BRCA* mutation were independent risk factors for poor PFS in patients receiving first‐line PARPi maintenance therapy for advanced ovarian cancer.

It is well known that *BRCA* mutation itself is a potent and favorable prognostic factor and is associated with high sensitivity to platinum‐based regimens as well as PARPi in ovarian cancer patients.^14^, ^15^, ^16^ Consistently, our study demonstrated that the absence of a *BRCA* mutation was one of the significant risk factors for a poor PFS. Fu et al. reported that PDS stage 3, compared to stage 4, and no gross residual lesion after debulking surgery were associated with favorable prognosis in patients with germline *BRCA* mutations.^17^ As the above‐mentioned study focused on *BRCA* mutation status and excluded patients who received first‐line maintenance treatment, the impact of first‐line maintenance PARPi was not evaluated. Our subgroup analysis of patients with *BRCA* mutations showed that high serum CA‐125 levels before starting PARPi were the only independent risk factor for a poor PFS. This finding is consistent with that of a subgroup analysis of SOLO 1.^18^ This study demonstrated that patients who underwent surgery with no gross residual tumor and had a complete response after platinum‐based chemotherapy were more likely to benefit from first‐line PARPi maintenance than those who had residual tumors and a partial response to adjuvant chemotherapy, respectively. These results suggest that first‐line PARPi maintenance might be more effective in patients with low tumor loads than in those with high tumor loads, which is believed to be related to the synthetic lethality and antitumor mechanism of PARPi.^19^ Synthetic lethality is where the loss of one gene is compatible with cell viability; however, simultaneous disruption of two genes results in cell death.^20^ Because of its synthetic lethality, the antitumor effect of PARPi is thought to be lower than that of conventional chemotherapeutic drugs, and PARPi is mainly recommended as maintenance therapy for ovarian cancer.^19^ Interestingly, tumor burden only at the time of starting PARPi was a significant risk factor in our study; however, other factors reflecting overall tumor burden, such as stage, pretreatment CA‐125 levels, and postoperative gross residual tumor, were not. This finding suggests that additional cycles of platinum‐based adjuvant chemotherapy causing further reduction in the pre‐PARPi tumor burden could benefit ovarian cancer patients with *BRCA* mutations.

In contrast, our study showed that in patients without *BRCA* mutations, non‐HGSC histology, and NAC were independent poor prognostic factors for PFS. Non‐serous histological type is known to be associated with a poor prognosis in advanced ovarian cancer.^21^ The deleterious impact of low sensitivity to platinum‐based chemotherapy and PARPi might be substantial in women without *BRCA* mutations, particularly in non‐HGSC patients.^22^

In the current study, the recurrence rate was 20.9% in patients with first‐line PARPi maintenance treatment during a relatively short median follow‐up period from the start of PARPi of 10 months. This is consistent with the results of SOLO1 and PRIMA studies. One‐year progression rates in these studies were 12% and 35%, respectively.^6^, ^8^ Fewer stage IV disease (15% vs. 33%) and higher rate of *BRCA* mutation (100% vs. 63.9%) in SOLO1 than in our study were noted.^6^ More patients received NAC as primary treatment in PRIMA studies than in our study (63% vs. 37%).^8^ These differences in the study populations may explain the differences in recurrence rates between our study and previous studies.

Of 40 patients in the recur/PD group, 38 (95.0%) stopped taking PARPi because of recurrence or PD during PARPi use. Thirty‐two (84.2%) recur/PD group cases had recurrence or PD within 1 year of PARPi use. SOLO1 and PRIMA studies lacked data regarding the timing of recurrence or PD after PARPi use. Our data will help clinicians manage patients.

Tumor biology and treatment strategies for newly diagnosed and recurrent ovarian cancers are different.^23^ Randomized controlled trials on PARPi demonstrated greater survival benefit in first‐line maintenance settings than in second‐line or more maintenance settings.^6^, ^8^, ^24^, ^25^ In line with this, the risk factors for poor PFS in the first‐line maintenance of PARPi are thought to be different from those in the second‐line or beyond. However, the existing studies of risk factors for poor survival during PARPi maintenance therapy focused on second‐line or more settings in recurrent ovarian cancer.^26^ A previous meta‐analysis has shown that *BRCA* mutation, homologous recombination deficiency (HRD)‐positive status, and sensitivity to platinum‐based chemotherapy are the factors indicating favorable prognosis in patients using PARPi. In contrast, the response to platinum‐based chemotherapy, surgery type, residual disease after surgery, stage, and age could not predict the efficacy of PARPi use.^15^ This meta‐analysis included prospective studies on every treatment setting for PARPi use, such as first‐line, second‐line, or more maintenance settings. Therefore, the risk factors for a poor PFS in the first‐line PARPi maintenance setting could not be identified.^26^ Our study focused on first‐line PARPi maintenance treatment.

The strength of this study is that it is the first to focus on risk factors for the failure of first‐line PARPi maintenance therapy in real‐world clinical settings and to identify whether these risk factors differ according to *BRCA* mutation. In addition, the information of a modest number of study participants was obtained by collecting data from six large university hospitals. However, this study had some limitations as well. First, it may have a potential bias because of its retrospective nature. Second, the analysis of various genetic mutations other than *BRCA* mutations could not be performed because the protocols and methods of genetic testing were different for each institution. In most patients, the HRD test could not be performed because of the high cost and the insurance system in Korea. Finally, this study had a relatively short follow‐up period to analyze the OS or outcomes of subsequent treatment in patients who experienced recurrence or progression after first‐line PARPi maintenance therapy.

In conclusion, non‐HGSC histology, NAC, high serum CA‐125 levels before starting PARPi, and no *BRCA* mutation might be risk factors for early failure of first‐line PARPi maintenance therapy in patients with advanced ovarian cancer. However, in women with *BRCA* mutations, pre‐PARPi high serum CA125 levels, which represent a high tumor burden before PARPi, were the only independent risk factor for a poor PFS. Non‐HGSC histology and NAC as primary treatments were poor prognostic factors associated with PFS in patients without *BRCA* mutations. Long‐term follow‐up data and further studies focusing on various genetic mutations, including HRD, are required. In addition, further studies on the PARPi treatment‐free interval and PARPi‐sensitive or ‐resistant recurrence after completion of PARPi use are required.

## AUTHOR CONTRIBUTIONS

**Nam Kyeong Kim:** Conceptualization (equal); data curation (equal); formal analysis (lead); methodology (equal); visualization (lead); writing – original draft (lead); writing – review and editing (equal). **Yeorae Kim:** Data curation (equal); investigation (equal); writing – review and editing (equal). **Hee Seung Kim:** Investigation (equal); supervision (equal); writing – review and editing (equal). **Soo Jin Park:** Investigation (equal); writing – review and editing (equal). **Dong Won Hwang:** Data curation (equal); investigation (equal); writing – review and editing (equal). **Sung Jong Lee:** Investigation (equal); supervision (equal); writing – review and editing (equal). **Ji Geun Yoo:** Data curation (equal); investigation (equal); writing – review and editing (equal). **Suk‐Joon Chang:** Investigation (equal); supervision (equal); writing – review and editing (equal). **Joo‐Hyuk Son:** Data curation (equal); investigation (equal); writing – review and editing (equal). **Tae‐Wook Kong:** Investigation (equal); supervision (equal); writing – review and editing (equal). **Jeeyeon Kim:** Data curation (equal); investigation (equal); writing – review and editing (equal). **Seung‐Hyuk Shim:** Investigation (equal); supervision (equal); writing – review and editing (equal). **A Jin Lee:** Data curation (equal); investigation (equal); writing – review and editing (equal). **Dong Hoon Suh:** Conceptualization (equal); formal analysis (equal); methodology (equal); project administration (lead); supervision (equal); visualization (equal); writing – review and editing (lead). **Yoo‐Young Lee:** Investigation (equal); supervision (lead); writing – review and editing (lead).

## FUNDING INFORMATION

Not applicable.

## CONFLICT OF INTEREST STATEMENT

The authors declare that there are no conflicts of interest.

## ETHICS STATEMENT

All six institutions participating in this study were approved by the Institutional Review Board (Seoul National University Bundang Hospital, Seoul National University Hospital, Samsung Medical Center, St. Mary's Hospital, Ajou University School of Medicine, and Konkuk University School of Medicine). The study was conducted in accordance with the principles of the Declaration of Helsinki.

## CONSENT

Informed consent was waived due to the retrospective nature of the review.

## Supporting information


**Table S1.** Clinicopathologic factors according to cancer recurrence or progression in patients with *BRCA* mutation
Table S2. Clinicopathologic factors according to cancer recurrence or progression in patients without *BRCA* mutation

## Data Availability

The data that support the findings of this study are available from the corresponding author upon reasonable request.
