# Supplementary material for: Risk factors for the failure of first‐line PARP inhibitor maintenance therapy in patients with advanced ovarian cancer: Gynecologic Oncology Research Investigators Collaboration Study (GORILLA‐3004)
Source: Cancer Med. 2023 Sep 28;12(19):19449–59. doi: 10.1002/cam4.6546 (PMC10587974; doi:10.1002/cam4.6546)
Supplement: Supplementary file 1 — Table S1. Clinicopathologic factors according to cancer recurrence or progression in patients with BRCA mutation Table S2. Clinicopathologic factors according to cancer recurrence or progression in patients without BRCA mutation [file CAM4-12-19449-s001.docx]

| Supplementary Table 1. Clinicopathologic factors according to cancer recurrence or progression in patients with *BRCA* mutation | | | |
| --- | --- | --- | --- |
|  | Non-recur/PD group  (n=101) | Recur/PD group  (n=20) | P |
| Age at diagnosis (years) | 57.7 ± 9.9 | 57.9 ± 9.7 | 0.961 |
| < 58 | 51 (50.5) | 11 (55.0) | 0.713 |
| ≥ 58 | 50 (49.5) | 9 (45.0) |  |
| Initial CA-125 at diagnosis (U/ml) | 1891.5 ± 3183.8 | 1775.7 ± 2135.8 | 0.877 |
| < 1872.3 | 76 (75.2) | 13 (65.0) | 0.342 |
| ≥ 1872.3 | 25 (24.8) | 7 (35.0) |  |
| CA-125 before PARPi (U/ml) | 24.0 ± 72.1 | 38.9 ± 61.6 | 0.389 |
| < 26.4 | 86 (85.1) | 13 (65.0) | 0.033 |
| ≥ 26.4 | 15 (14.9) | 7 (35.0) |  |
| Total cycle number of chemotherapy before PARPi | 6.7 ± 1.6 | 7.0 ± 1.8 | 0.550 |
| FIGO stage |  |  | 0.392 |
| III | 56 (55.4) | 9 (45.0) |  |
| IV | 45 (44.6) | 11 (55.0) |  |
| Postoperative gross residual disease |  |  | 0.032 |
| No | 62 (63.3) | 7 (36.8) |  |
| Yes | 36 (36.7) | 12 (63.2) |  |
| Histology |  |  | 0.589 |
| HGSC | 96 (95.0) | 20 (100.0) |  |
| Non-HGSC | 5 (5.0) | 0 (0.0) |  |
| Type of PARPi |  |  | 0.761 |
| Olaparib | 62 (61.4) | 13 (65.0) |  |
| Niraparib | 39 (38.6) | 7 (35.0) |  |
| Duration of PARPi use (months) | 12.3 ± 7.3 | 7.1 ± 5.3 | 0.003 |
| Primary treatment |  |  | 0.343 |
| PDS | 62 (61.4) | 10 (50.0) |  |
| NAC followed by IDS* | 39 (38.6) | 10 (50.0) |  |
| Values are presented as mean ± standard deviation or n (%).  * Patients who underwent palliative chemotherapy without surgery were included.  FIGO, International Federation of Gynecology and Obstetrics; HGSC, high-grade serous carcinoma; IDS, interval debulking surgery; NAC, neoadjuvant chemotherapy; PARPi, Poly (ADP-ribose) polymerase inhibitor; PD, progression of disease; PDS, primary debulking surgery | | | |

| Supplementary Table 2. Clinicopathologic factors according to cancer recurrence or progression in patients without *BRCA* mutation | | | |
| --- | --- | --- | --- |
|  | Non-recur/PD group  (n=50) | Recur/PD group  (n=20) | P |
| Age at diagnosis (years) | 55.1 ± 9.7 | 58.6 ± 11.3 | 0.196 |
| < 56 | 28 (56.0) | 7 (35.0) | 0.112 |
| ≥ 56 | 22 (44.0) | 13 (65.0) |  |
| Initial CA-125 at diagnosis (U/ml) | 1394.5 ± 3132.7 | 896.3 ± 1095.3 | 0.502 |
| < 1257.4 | 39 (78.0) | 17 (89.5) | 0.491 |
| ≥ 1257.4 | 11 (22.0) | 2 (10.5) |  |
| CA-125 before PARPi (U/ml) | 14.2 ± 26.9 | 29.3 ± 38.1 | 0.124 |
| < 18.3 | 45 (90.0) | 13 (68.4) | 0.029 |
| ≥ 18.3 | 5 (10.0) | 6 (31.6) |  |
| Total cycle number of chemotherapy before PARPi | 6.6 ± 1.3 | 6.7 ± 1.6 | 0.934 |
| FIGO stage |  |  | 0.214 |
| III | 33 (66.0) | 10 (50.0) |  |
| IV | 17 (34.0) | 10 (50.0) |  |
| Postoperative gross residual disease |  |  | 0.182 |
| No | 30 (60.0) | 8 (42.1) |  |
| Yes | 20 (40.0) | 11 (57.9) |  |
| Histology |  |  | 0.027 |
| HGSC | 46 (92.0) | 14 (70.0) |  |
| Non-HGSC | 4 (8.0) | 6 (30.0) |  |
| Type of PARPi |  |  | >0.999 |
| Olaparib | 2 (4.0) | 0 (0.0) |  |
| Niraparib | 48 (96.0) | 20 (100.0) |  |
| Duration of PARPi use (months) | 8.4 ± 6.1 | 5.5 ± 3.9 | 0.021 |
| Primary treatment |  |  | 0.020 |
| PDS | 35 (70.0) | 8 (40.0) |  |
| NAC followed by IDS* | 15 (30.0) | 12 (60.0) |  |
| Values are presented as mean ± standard deviation or n (%).  * Patients who underwent palliative chemotherapy without surgery were included.  FIGO, International Federation of Gynecology and Obstetrics; HGSC, high-grade serous carcinoma; IDS, interval debulking surgery; NAC, neoadjuvant chemotherapy; PARPi, Poly (ADP-ribose) polymerase inhibitor; PD, progression of disease; PDS, primary debulking surgery | | | |
